# New Benzofuran Derivatives as an Antioxidant Agent

**DOI:** 10.4103/0250-474X.65022

**Published:** 2010

**Authors:** S. S. Rindhe, M. A. Rode, B. K. Karale

**Affiliations:** Department of Chemistry, New Arts Commerce and Science College, Ahmednagar- 414 001, India; 1 Department of Chemistry, Radhabai Kale Mahila Mahavidyalaya, Ahmednagar-414 001, India

**Keywords:** Benzofurans, antimicrobial activity, antioxidant activity

## Abstract

A series of substituted benzofuran derivatives were synthesized and characterized by spectral data. Some of the synthesized compounds were tested for *in vitro* antioxidant activity. Some of them have shown very good antioxidant activity. These compounds were also tested for antimicrobial activity against microbial strains viz. *staphylococcus aureus* (NCIM 5021) and *salmonella typhimurium* (NCIM 2501), but none of them showed any activity against these microorganisms.

Various natural and synthetic benzofuran derivatives are found to possess diverse applications in the field of medicine. A crystalline antioxidant extracted from yeast was shown to protect *in vitro* the erythrocytes of vitamin E deficient rats from hemolysis. The structure of this compound was determined as benzofuran derivative[1]. It is well known that Vitamin E having chroman skeleton have a very good antioxidant activity. It has been reported that the activity is increased by the transformation of skeleton from chroman to benzofuran[2]. The antioxidant activity of a novel water soluble antioxidant of the benzofuran family (5-hydroxy-4,6,7-trimethyl-2,3-dihydrobenzofuran-2-acetic acid, BFA) is reported to possess better antioxidant activity than that of congener compound Trolox C[3].

Benzofuran in conjunction with cyclic β-amino hydroxamic acid scaffolds have found to possess very potent and selective tumor necrosis factor-ά converting enzyme (TACE) inhibitory activity. Similarly some of the benzofuran derivatives have shown very good antimicrobial activity and β-amyloid aggregation inhibitory activity[4–6]. Owing to the biological importance of benzofurans, we herein, report the synthesis and biological testing of some benzofurans.

In the present work, substituted phenacyl bromide (1) was treated with 2'-hydroxy-5'-nitro acetophenone (2) in presence of K_2_CO_3_ in DMF to get aryl-3-methyl-5-nitro-1-benzofuran-2-ylmethanone (3), which was further reduced to aryl-5-amino-3-methyl-1-benzofuran-2-ylmethanone (4). Compound 4 was further treated with 5-chloronicotinoyl chloride to get N-(2-aroyl)-3-methyl-1-benzofuran-5-yl)-6-chloronicotinamide (5). Compound 5 was treated with various substituted amines in pyridine to get benzofuran derivatives 6(a-r), respectively.

All recorded melting points were determined in open capillary tubes and were uncorrected. IR spectra were recorded on Perkin-Elmer FTIR spectrophotometer in KBr disc.^1^H NMR spectra were recorded on 400 MHz spectrophotometer in DMSO-d_6_ as a solvent and TMS as an internal standard. Peak values are shown in δ ppm. Mass spectra were obtained by Waters mass spectrometer.

General procedure employed for the preparation of aryl-3-methyl-5-nitro-1-benzofuran-2-ylmethanone (3) was equimolar mixture of substituted (0.1mol) phenacyl bromide 1 and (0.1 mol) 2'-hydroxy-5'-nitro-acetophenone 2 with (0.3 mol) K_2_CO_3_ in DMF was heated at 80° for 5 h. The reaction mixture was cooled to room temperature and then poured into ice cooled water. The solid product was separated by filtration and crystallized from DCM-hexane.

The procedure for the preparation of aryl-5-amino-3-methyl-1-benzo furan-2-yl phenyl methanone (4) involved adding to a suspension of (0.1 mol) nitro derivative (3) in methanol (50 ml) 5 equivalents of SnCl_2_.2H_2_O and heating the reaction mixture at 60° for 4 h. The reaction mixture was cooled to room temperature. It was poured into liquid NH_3_ and filtered through hyflow. Filtrate was extracted by EtOAc. EtOAc layer separated, dried over Na_2_SO_4_ and concentrated under vacuum. The product was crystallized from ethanol.

N-(2-aroyl)-3-methyl-1-benzofuran-5-yl)-6-chloro nicotinamide (5) was prepared by stirring an equimolar mixture of (0.1 mol) amino compound (4) and (0.1 mol) 6-chloro nicotinoyl chloride in THF with (0.3 mol) K_2_CO_3_ for 6 h at room temperature. The reaction mixture was concentrated under vacuum and then poured into water. The solid product was separated by filtration and recrystallized from ethanol.

General procedure for the preparation of (6 a-r) involved adding a suspension of (0.1 mol) chloro compound 5 in pyridine to (0.12 mol) substituted amine. The suspension was heated for 12 h at 110°. The reaction mixture was then cooled to room temperature and poured into water. The solid product was separated by filtration and crystallized from DCM-Hexane. The compounds synthesized by the above procedure are listed in Table 1 and their structural data, spectral data and physical constant values are given in Table 1 and 2.

**TABLE 1:** STRUCTURAL AND ANALYTICAL DATA OF THE SYNTHESIZED BENZOFURAN DERIVATIVES
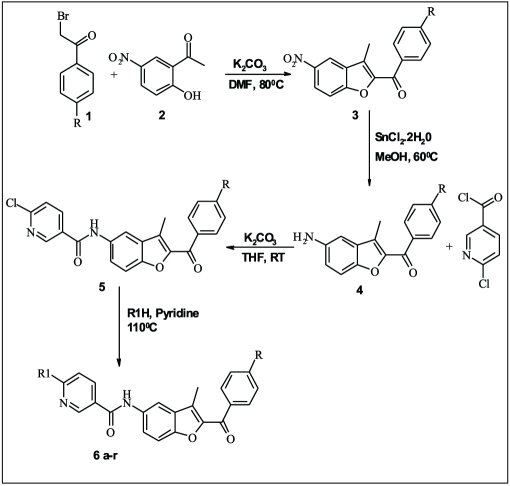

Compds.	R1	R	M.P. (°)	Yield (%)	Elemental analysis Calcd % (Found %)		
					C	H	N
6a	morpholine	Br	109-11	92	60.01 (60.00)	4.26 (4.25)	8.07 (8.05)
6b		OCH_3_	140-2	95	68.78 (68.77)	5.34 (5.33)	8.91 (8.90)
6c	N-methyl piperazine	Br	129-31	89	60.80 (60.79)	4.72 (4.71)	10.50 (10.49)
6d		OCH_3_	196-18	88	69.41 (69.40)	5.82 (5.81)	11.56 (11.55)
6e	N-benzyl piperazine	Br	118-20	95	65.03 (65.02)	4.80 (4.79)	9.19 (9.18)
6f		OCH_3_	119-21	90	72.84 (72.83)	5.75 (5.74)	9.99 (9.98)
6g	thiomorpholine	Br	147-9	59	58.21 (58.20)	4.13 (4.12)	7.83 (7.81)
6h		OCH_3_	135-7	55	66.51 (66.50)	5.17 (5.17)	8.62 (8.61)
6i	pyrrolidine	Br	128-30	69	61.91 (61.90)	4.40 (4.39)	8.33 (8.31)
6j		OCH_3_	126-8	67	71.19 (71.20)	5.53 (5.52)	9.22 (9.20)
6k	piperidine	Br	119-21	75	62.56 (62.55)	4.67 (4.66)	8.11 (8.10)
6l		OCH_3_	107-9	78	71.63 (71.62)	5.80 (5.79)	8.95 (8.94)
6m	tetrahydroisoquinoline	Br	131-3	88	65.73 (65.72)	4.27 (4.28)	7.42 (7.41)
6n		OCH_3_	139-41	80	74.26 (74.25)	5.26 (5.25)	8.12 (8.10)
6o	1-pyridyl-2-ylpiperazine	Br	218-20	76	62.42 (64.41)	4.39 (4.38)	11.74 (11.73)
6p		OCH_3_	174-6	66	70.19 (70.18)	5.34 (5.33)	12.79 (12.77)
6q	1,4-dioxa-8-azaspiro[4,5] decane	Br	137-9	60	60.43 (60.42)	4.55 (4.54)	7.29 (7.28)
6r	2-piperzin-1-ylethanol	OCH_3_	102-4	55	67.69 (67.68)	5.88 (5.87)	10.89 (10.88)

**TABLE 2:** SPECTRAL DATA OF SYNTHESIZED BENZOFURAN DERIVATIVES

Compd.	IR (cm-1)	Spectral data 1H NMR δ (ppm)	Mass M+
6a	1644, 2922, 1289, 3316,	2.58 (3H, s), 3.61 (4H, s), 3.85 (4H, s), 6.94-8.32 (10H, m), 10.22 (1H, s)	520
6b	1635, 2957, 1261,1166, 3301	2.55 (3H, s), 3.61 (4H, d), 3.72 (4H, d), 3.88 (3H, s), 6.93-8.80 (10H, m), 10.21 (1H, s)	471
6c	1634, 2934, 1298, 3287	2.22 (3H, s), 2.40 (4H, t) 2.57 (3H, s), 3.64 (4H, s), 6.93-8.77 (10H, m), 10.20 (1H, s)	533
6d	1634, 2934, 1298, 1168, 3287	2.39 (3H, s), 2.55 (3H, s), 2.64 (4H, s), 3.71(4H, d), 3.89 (3H, s), 6.96-8.79 (10H, m), 10.21 (1H, s)	484
6e	1644, 2922, 1289, 3316	2.50 (4H, s), 2.57 (3H, s), 3.53 (2H, s), 3.65 (4H, s) 6.92-8.76 (15H, m), 10.20 (1H, s)	609
6f	1644, 2922, 1289, 1168, 3316	2.45 (4H, t), 2.55 (3H, s), 3.53 (2H, s), 3.64 (4H, t), 3.88 (3H, s), 6.91-8.76 (15H, m), 10.18 (1H, s)	560
6g	1635, 2914, 1261, 3301	2.57 (3H, s), 2.63 (4H, s), 4.03 (4H, s), 7.66-8.32 (10H, m), 10.69 (1H, s).	536
6h	1635, 2914, 1261, 1166, 3301	2.56 (3H, s), 2.63 (4H, t), 3.87 (3H, s), 4.01 (4H, t), 6.95-8.78 (10H, m), 10.20 (1H, s)	487
6i	1642, 2933, 1291, 3301	1.98 (4H, s), 2.57 (3H, s), 3.47 (4H, s), 6.53-8.76 (10H, m), 10.13 (1H, s).	504
6j	1636, 2928, 1321, 3305	1.97 (4H, s), 2.55 (3H, s), 3.41(4H, s), 3.87 (3H, s), 6.53-8.77 (10H, m), 10.13 (1H, s)	455
6k	1642, 2933, 1291, 3301	1.55 (4H, s), 1.63 (2H, m), 2.57 (3H, s), 3.47 (4H, s), 7.02-8.76 (10H, m), 10.16 (1H, s).	518
6l	1637, 2932, 1289, 3312	1.55 (4H, s), 1.64 (2H, m), 2.55 (3H, s), 3.66 (4H, s), 3.88 (3H, s), 7.10-8.76 (10H, m), 10.19 (1H, s)	469
6m	1635, 291, 1281, 3311	2.57 (3H, s), 2.93 (2H, s), 3.91 (2H, s), 4.81 (2H, s),7.21-8.82 (14H, m), 10.21 (1H, s).	566
6n	1636, 2933, 1291, 3320	2.55 (3H, s), 2.93 (2H, t), 3.84 (2H, t), 3.90 (3H, s), 4.82 (2H, t), 7.12-8.81 (14H, m), 10.20(1H, s)	517
6o	1644, 2933, 1288, 3300	2.51 (3H, s), 3.18 (4H, s), 3.69 (4H, s), 7.45-8.99 (14H, m), 10.70 (1H, s)	596
6p	1635, 2923, 1291, 3305	2.55 (3H, s), 3.28 (4H, s), 3.64 (2H, s), 3.77 (2H, s), 3.84 (3H, s), 6.63 - 9.00 (14H, m), 10.68 (1H, s)	547
6q	1645, 2945, 1288, 3320	1.65 (4H, t), 2.57 (3H, s), 3.76 (4H, t), 3.93 (4H, t), 6.98-8.78 (10H, m), 10.19 (1H, s).	576
6r	1645, 2930, 1290, 3306	2.44 (2H, t), 2.55 (3H, s), 3.38 (4H, t), 3.53 (2H, t), 3.63 (4H, t), 3.88 (3H, s), 6.92-8.77 (10H, m), 10.18 (1H, s).	514

A series of 18 new compounds were synthesized. The structural data of all benzofuran derivatives is shown in Table 1. Scheme 1 illustrates the preparation of target compounds. The structure of the synthesized compounds was elucidated by IR, ^1^H NMR, mass spectral data and elemental analysis.

**Scheme 1:**
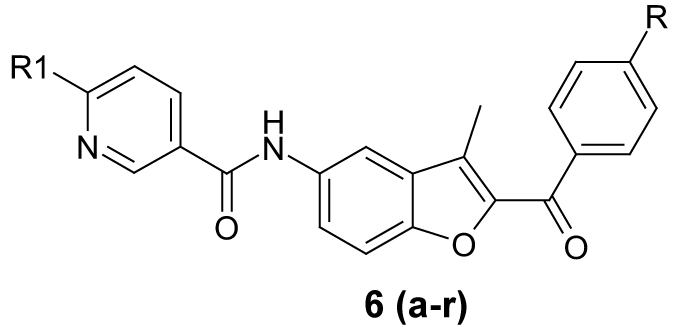
Synthetic route for the preparation of novel benzofuran derivatives General method of synthesis of N-(2-aroyl)-3-methyl-1-benzofuran-5-yl)-6-aminoalkylnicotinamide 6(a-r)

In the ^1^H NMR spectra of the compounds, NH proton of the benzofuran ring was seen as singlet at about 10.20-10.70 δ ppm. Signal due CH_3_ of benzofuran appeared at 2.55-2.58 δ ppm as a singlet. All other aromatic and aliphatic protons were observed in the expected regions. Mass spectra of all compounds showed M^+^ peaks in agreement with their molecular formula. In the IR spectra of all compounds C=O stretching bands were observed at 1635-1644 cm^−1^and stretching band of CH_3_ of benzofuran ring observed at 2914-2932 cm^−1^.

The benzofuran derivatives were evaluated for their *in vitro* antimicrobial activity. MICs were recorded as the minimum concentration of compound, which inhibit the growth of tested microorganisms. Antimicrobial activities of the compounds were tested using Mueller-Hinton broth (Hi Media M 391) medium. Microbial strains used for testing included, *Staphylococcus aureus* (NCIM 5021) and *Salmonella typhimurium* (NCIM 2501). All test compounds were found to be inactive against above bacterial strains.

The *in vitro* antioxidant activity of the test compounds was determined by DPPH method using L-ascorbic acid (an antioxidant agent) as a positive control. The compounds were tested for antioxidant activity at 200, 100 and 50 μg/ml concentration. Amongst the compounds screened for antioxidant activity, 6a, 6b, 6d, 6h, 6o, 6p and 6r showed very good antioxidant activity as shown in Table 3.

**TABLE 3:** % ANTIOXIDANT ACTIVITY OF THE COMPOUNDS

Concentration in μg/ml	200	100	50
L-ascorbic acid	99.2	99	98.8
6a	95.3	95.3	91.9
6b	100	98.3	88.1
6d	94.6	80.2	28.7
6g	57.9	48.0	28.2
6h	98.1	91	89.8
6o	97.9	94	85.7
6p	100	97.2	93.6
6r	94.2	93.2	92.6

The compounds with morpholine, 1-pyridyl-2-yl piperazine at R1 and with Br and OMe at R showed very significant antioxidant activity. While the compounds with thiomorpholine and piperzin-1-yl ethanol at R1 and OMe at R also showed good antioxidant activity. Compounds with pyrrolidine, piperidine and N-benzylpiperazine at R1 do not show any antioxidant activity.
